# Electrical pulse-induced electrochemical biosensor for hepatitis E virus detection

**DOI:** 10.1038/s41467-019-11644-5

**Published:** 2019-08-19

**Authors:** Ankan Dutta Chowdhury, Kenshin Takemura, Tian-Cheng Li, Tetsuro Suzuki, Enoch Y. Park

**Affiliations:** 1 Research Institute of Green Science and Technology, Shizuoka University, 836 Ohya, Suruga-ku, Shizuoka, 422-8529 Japan; 2 Department of Bioscience, Graduate School of Science and Technology, Shizuoka University, 836 Ohya, Suruga-ku, Shizuoka, 422-8529 Japan; 3 Department of Virology 2, National Institute of Infectious Diseases, 4-7-1 Gakuen, Musashimurayam-shi, Tokyo, 208-0011 Japan; 4 Department of Infectious Disease, Hamamatsu University of School of Medicine, Handayama, Hamamatsu, 431-3125 Japan

**Keywords:** Sensors and probes, Hepatitis, Health care, Biotechnology

## Abstract

Hepatitis E virus (HEV) is one of the leading causes of acute viral hepatitis worldwide. In this work, a pulse-triggered ultrasensitive electrochemical sensor was fabricated using graphene quantum dots and gold-embedded polyaniline nanowires, prepared via an interfacial polymerization and then self-assembly approach. Introducing an external electrical pulse during the virus accumulation step increases the sensitivity towards HEV due to the expanded surface of the virus particle as well as the antibody-conjugated polyaniline chain length, compared to other conventional electrochemical sensors. The sensor was applied to various HEV genotypes, including G1, G3, G7 and ferret HEV obtained from cell culture supernatant and in a series of fecal specimen samples collected from G7 HEV-infected monkey. The sensitivity is similar to that detected by real-time quantitative reverse transcription-polymerase chain (RT-qPCR). These results suggests that the proposed sensor can pave the way for the development of robust, high-performance sensing methodologies for HEV detection.

## Abstract (web summary)

Detection of viral biomarkers is important for disease treatment and prevention. Here, the authors report on a system that uses an electrical pulse-induced electrochemical sensor for the detection of hepatitis E virus, and demonstrate potential application of the device.

## Introduction

Viruses are a major cause of human diseases in populated countries such as China and India, as well as in developing countries with remote settings and limited resources. Fast and early detection should be one of the best ways to prevent an outbreak and requires the development of a biosensor that would ideally produce a quantitative signal for individual viral particles^1,2^. To achieve such a biosensor with high accuracy, the development of promising biosensing materials and sensing strategies has become one of the most important issues in current science^3–6^. Among recently evolved viral diseases where a low concentration of virus could be a potentially fatal threat, hepatitis E virus (HEV) infection is primarily known to cause acute hepatitis. The four major HEV genotypes (G1–G4) are known to be endemic in many industrialized countries, particularly in Japan and Europe^7^. Every year, over 20 million people worldwide are infected with HEV, and over 3 million people develop hepatitis E^8^. In addition, HEV infection in pregnant women is more likely to cause fulminant hepatitis. According to WHO’s estimation, the death toll from HEV infection was 44,000 in 2015^9^, which indicates that the HEV could be more widespread in society than was originally thought^10,11^.

For clinical diagnosis of HEV infection, detection techniques of anti-HEV antibodies and the viral RNAs mainly based on enzyme immunoassay and reverse transcription-polymerase chain reaction (RT-PCR), respectively, are widely used at present^11^. IgM and IgA anti-HEV antibodies are key markers of the acute viral infection. Very high titers of IgG anti-HEV antibody can also be a surrogate marker of the acute infection. However, their clinical utility to indicate the current HEV infection is limited because of relatively low sensitivity and/or short-lived^10^. RT-PCR methods are sensitive and specific. However, availability of diagnostic PCR assays still remains limited because they are costly and require highly skilled professional for operation not to yield false-positive results due to contamination of the reactions with laboratory-amplified nucleic acids. Although it is generally accepted that antigen detection methods are useful for clinical diagnosis of viral infection, sensitive and reliable methods for detection of HEV antigen have not been established to date. Therefore, the development of sensitive and reliable techniques for the detection of HEV in food as well as patient samples is urgently needed to ensure health safety.

The continuous development of nanotechnology offers new horizons for electrochemical analysis, especially in impedimetric sensing. Applying the most accepted antibody–antigen interaction, several nanomaterials are already used to fabricate the immunosensors, including nanocomposites such as quantum dots^12–14^, metal nanoparticles^15–18^, and conducting polymers^19–22^ to amplify the detection signal. In the quest for superior performance, most research has focused on developing a better nanostructured matrix for entrapping more analytes in a single step with high specificity. Unfortunately, this approach seems to have reached saturation. Therefore, instead of developing the base matrix, in this report, we have focused on engineering the impedimetric process, inducing pulse generation. To initiate, we fabricated a biosensor electrode using specific anti-HEV antibody-conjugated to nitrogen- and sulfur-codoped graphene quantum dots (Ab-N,S-GQDs) and gold-embedded polyaniline nanowires (AuNP-PAni) as the electrode matrix. As well-studied nanostructured conducting polymers such as polyaniline exhibit long-term stability and offer routes to influence the interactions between matrix and nanoparticles^19,22^, we have chosen polyaniline as the base material. On the other hand, the noble metal in Au nanoparticles (AuNPs) with well-defined and controlled shapes has attracted increasing attention and become a promising material for the ultrasensitive detection of chemical and biological molecules^23–25^. As a promising material in the carbon family, GQDs have the most prospective applications in synthesizing nanocomposites and constructing biosensors^13,26^. According to previous research, the structural defects of GQDs may be manipulated by doping heteroatoms into the π-conjugated carbon system, generating useful functionality for nanocomposite formation^27,28^. In N,S-GQDs, the chemically bonded nitrogen atom could drastically enhance the electrochemical properties by altering the electronic characteristics, and the sulfur can increase the number of anchoring sites for adsorption onto noble nanoparticles. In addition, N,S-GQDs could be used to amplify electrochemical activity and conjugate antibodies via their edge carboxylic groups^29^. By combining these three components, PAni, AuNPs, and N,S-GQDs, a nanocomposite was created here that possesses the merits of both its organic and inorganic components and may also exhibit different properties that one component does not have. The N,S-GQDs can form covalent conjugates with the antibody, which is embedded with Au-PAni (Ab-N,S-GQDs@AuNP-PAni) nanocomposites. The nanocomposites hence show excellent electroactivity in analyte solution, which can be applied for the application of virus detection by impedimetric processes.

**Fig. 1:**
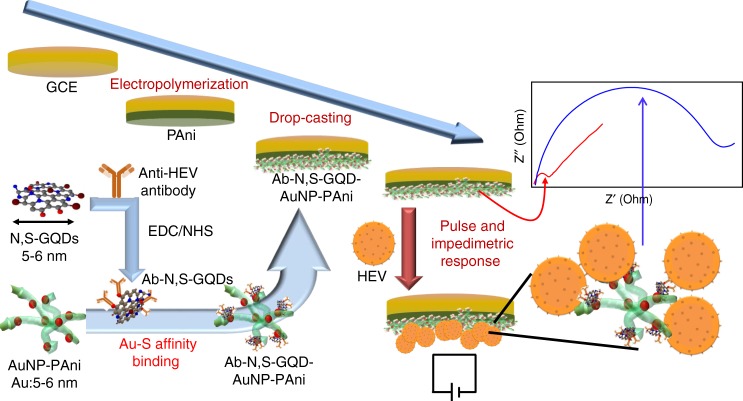
Schematic diagram of the Ab-N,S-GQDs@AuNP-PAni nanocomposite-loaded sensor electrode and its pulse-induced impedimetric sensing of HEV

In this report, the nanocomposites were deposited on a finely electropolymerized polyaniline-coated GCE to form an Ab-N,S-GQDs@AuNP-PAni/PAni||GCE sensor, and HEV was detected by an impedimetric response (Fig. 1). To achieve high sensitivity at low virus concentrations, this biosensor was applied with different external pulses during the virus loading process, which was optimized to achieve the best results at +0.8 V (positive with respect to Ag/AgCl reference electrode). Under this optimal condition, the proposed biosensor demonstrates its ability to detecting HEV in a wide linear range with a low detection limit. The specificity and sensitivity of the proposed biosensor has been tested subsequently in HEV-like particles (HEV-LPs) in buffer and then human serum. In addition, the HEV samples that collected from cell culture supernatant and fecal specimens of HEV-infected monkey were used to confirm its applicability.

## Results

### Characterization of N,S-GQDs@AuNP-PAni nanocomposites

**Fig. 2:**
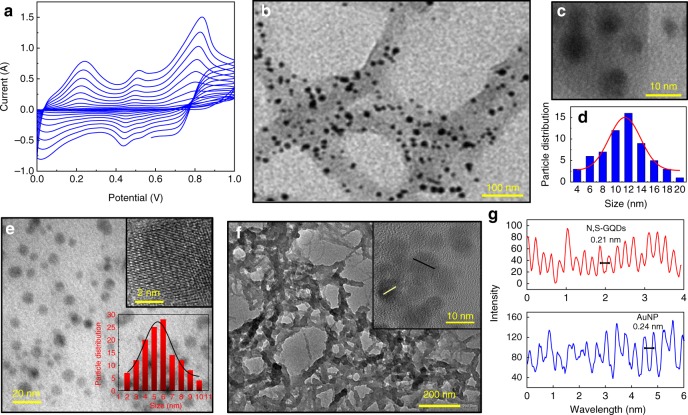
Characterizations of polyaniline deposition, N,S-GQDs and Au-PAni preparation and N,S-GQDs@AuNP-PAni nanocomposites: **a** cyclic voltammograms of electropolymerized PAni film on the GCE electrode, **b** TEM image of AuNP-PAni nanocomposites, **c** high magnification of AuNP on the PAni chain, **d** size distribution of the AuNPs inside the AuNP-PAni nanocomposites, **e** TEM image of N,S-GQDs (inset: HRTEM image of an isolated N,S-GQD and the size distribution), **f** TEM image of N,S-GQDs@AuNP-PAni nanocomposites (inset: HRTEM of a small area), and **g** fringe patterns of N,S-GQDs and AuNP from the HRTEM image of N,S-GQDs@AuNP-PAni nanocomposites

To make a suitable electrochemical matrix, a fine layer of bare polyaniline film was synthesized on a clean GCE by electrochemical polymerization. Figure 2a illustrates cyclic voltammograms for the synthesis of polyaniline on GCE for 10 cycles, carried out using 0.1 M aniline in 0.5 M H_2_SO_4_ in the potential range between 0 and 1 V. Three peaks at 0.18, 0.49, and 0.77 V represent the characteristic peaks for the idealized redox processes of leucoemeraldine to emeraldine and emeraldine to pernigraniline forms of polyaniline^21^. The film was deposited on the electrode to make an adhesive platform for anchoring the drop-casted N,S-GQDs@AuNP-PAni nanocomposites for the next step^30^. As the polished GCE electrode surface is very smooth, there is some possibility of leaching of the drop-casted AuNP-PAni out during the electrochemical analysis. Being a porous material, the electropolymerized polyaniline layer has much more adhesive tendency than the bare electrode surface which can entrap the Au-PAni-N,S-GQDs more tightly. In addition, the positively charged background of polyaniline can also enhance the Au-PAni-N,S-GQDs entrapment via electrostatic interaction. The stability of the GCE||PAni/AuNP-PAni electrode has been investigated in CV over 50 cycles (Supplementary Fig. 1) where it can be noted that the electroactivity of the electrode remains almost unchanged even after its 50th cycle. Well-formed AuNP-PAni nanocomposites with exposed surface area were synthesized via interfacial self-oxidation-reduction polymerization, where only the aniline molecules at the interface of aqueous and organic layers were exposed to the HAuCl_4_ oxidant and underwent controlled polymerization (Supplementary Fig. 2)^31^. This method prevents branching of the polyaniline nanowires and arrests the polymer in its nanoscopic form. At the same time, Au^3+^ in the aqueous phase was reduced to its Au^0^ state and deposited onto the polyaniline nanowires with a homogeneous distribution.

In this work, AuNPs were selected for their easy immobilization and electrical wiring of N,S-GQDs within the modified electrode. Polyaniline nanowires with diameters of 50–70 nm are observed in the TEM image (Fig. 2b, c). The most interesting information in the high-magnification image in Fig. 2c is that the AuNPs are evenly dispersed within the polymer, and their size range is 6–14 nm with an average size of 11 ± 0.5 nm (Fig. 2d). As the N,S-GQDs have to be attached on the surface of nanocomposites via the gold thiol interaction, a dispersed distribution of the AuNPs is the most desirable result of this nanocomposite synthesis, as it can facilitate the increased loading of anti-HEV antibody-loaded N,S-GQDs. Prior to the attachment of the N,S-GQDs, the monoclonal anti-HEV antibody was covalently linked with the N,S-GQDs via EDC/NHS covalent chemistry^32^.

To get the best results, excess amount of N,S-GQDs and antibody has been introduced to AuNP-PAni to get maximum conjugation. However, to make an estimation of the loading, we have carried out the thermogravimetric analysis (TGA) and calculated the approximate loading percentage. From the degradation amount of the N,S-GQD and N,S-GQD-Ab-loaded AuNP-PAni nanocomposites, it can be noted that the 30 μg mg^−1^ of N,S-GQDs and 40 μg mg^−^^1^ of antibodies are bound in AuNP-PAni nanocomposites (Supplementary Fig. 3). The conjugation of the antibodies toward the HEV-LP was confirmed by ELISA, as shown in Supplementary Fig. 4. The TEM image of pristine N,S-GQDs is shown in Fig. 2e, where the homogeneously distributed dots confirm its formation. The particle size distribution and high-resolution TEM image are also shown in the inset of Fig. 2e. The characteristic fluorescence properties of the as-synthesized N,S-GQDs at 460 nm are also shown in Supplementary Fig. 5 for further confirmation^28^. After the thiol conjugation of the Ab-N,S-GQDs with AuNP-PAni nanocomposites, the nanowire formation still maintains its pristine nanotube structure with additional thickness due to the incorporation of N,S-GQDs (Fig. 2f). The high-resolution transmission electron microscopy (HRTEM) image of the N,S-GQDs@AuNP-PAni nanocomposites clearly shows two distinct fringe patterns of these two crystallized structures of N,S-GQDs and AuNPs, as illustrated in Fig. 2g. The characteristic fringe of 0.24 nm for AuNP^33^ can be distinguished immediately adjacent to the N,S-GQDs with a fringe distance of 0.21 nm, which is the characteristic fringe of the graphitic lattice^30^.

**Fig. 3:**
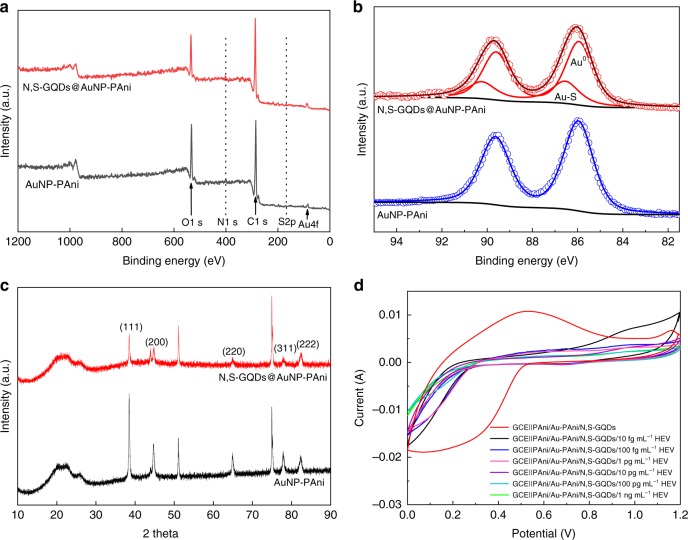
Characterizations of N,S-GQDs@AuNP-PAni nanocomposites: **a** survey spectrum of XPS, **b** deconvoluted Au 4f spectra, **c** XRD of N,S-GQDs@AuNP-PAni nanocomposites before and after attachment of N,S-GQDs, and **d** cyclic voltammetry (CV) of the Ab-N,S-GQDs@AuNP-PAni/PAni||GCE sensor electrode before and after the addition of different virus concentrations

The chemical attachment of N,S-GQDs on the AuNP-PAni nanocomposites was confirmed by XPS analysis (Fig. 3a). The survey spectrum of N,S-GQDs@AuNP-PAni shows the standard peaks of C 1s (284 eV), O 1s (531 eV), and Au 4f (85 eV) along with two additional peaks of N 1s (400 eV) and S 2p (163 eV). Although there was a slight N 1s peak already present in bare AuNP-PAni nanocomposites because of the nitrogen atom of aniline, its contribution increased significantly after conjugation with N,S-GQDs due to nitrogen doping. Similarly, the introduction of the small S 2p peak indicates the presence of N,S-GQDs on the N,S-GQDs@AuNP-PAni nanocomposites. To explore the thiol linkage between N,S-GQDs and Au-PAni, the Au 4f peaks are further deconvoluted and are shown in Fig. 3b. The Au 4f peak appears at 85.85 eV in the case of AuNP-PAni but is slightly shifted to 85.95 eV in the nanocomposite, indicating the presence of a more electronegative environment due to the adjacent sulfur^34^. In AuNP-PAni, the Au^0^ peak is clearly the sole contribution. However, the deconvoluted spectrum of Au 4f in N,S-GQDs@AuNP-PAni clearly introduces the contribution of an additional peak at 86.4 eV, confirming the formation of Au–S bonds. Similarly, the deconvolution of the S 2p spectra also confirms similar effects of Au–S formation (Supplementary Fig. 6).

To confirm the structural properties of the N,S-GQDs@AuNP-PAni nanocomposites, XRD analysis was carried out and is presented in Fig. 3c. Characteristic AuNP peaks are clearly visible at 2*θ* = 38.2°, 44.3°, 64.4°, 77.6°, and 81.8°, corresponding to the (111), (200), (220), (311), and (222) planes, respectively^35^. Due to the presence of polyaniline nanowires, a broad peak has also been observed at 2*θ* = 24°, revealing the amorphous nature of the carbon polymer chain^36^. A strong peak at 2*θ* = 76° is attributed to the stainless steel sample matrix and can be ignored for this analysis. After the formation of N,S-GQDs@AuNP-PAni, all the Au characteristic peaks remain in exactly the same positions with relatively decreasing peak intensities. The addition of the carbon-enriched N,S-GQDs to the nanocomposites does not affect the crystal pattern of the AuNPs even after gold thiol bonding; thus, the electroactive nature of the nanocomposites is maintained in their use for electrochemical sensing.

To investigate the electroactivity of the nanocomposites on the GCE (Ab-N,S-GQDs@AuNP-PAni/PAni||GCE) before proceeding to HEV detection, CVs were recorded for each immobilization step at a scan rate of 100 mV s^−1^ (Fig. 3d). The bare sensor electrode (Ab-N,S-GQDs@AuNP-PAni/PAni||GCE) without any virus loading shows a wide spectrum, indicating the superb electroactivity of the sensor. A clear redox peak is observed at +0.45/+0.16 V due to the most electroactive form of the emeraldine salt of polyaniline^21,37^. However, a drastic decrease in peak current was observed when the initial virus solution was tested on the sensor electrode. This decrease may be because the addition of virus solution not only creates hindrance of the electronic movement of the electrode but also increases solution resistance (*R*_sol_), decreasing the overall electroactivity. Thereafter, the current intensities decreased gradually when increasing concentrations of the viruses were added onto the surface of the electrode, which indicates that the attachment of virus molecules restricted the electron transfer process^38–40^.

### Impedimetric response and its effect on electrical pulse

**Fig. 4:**
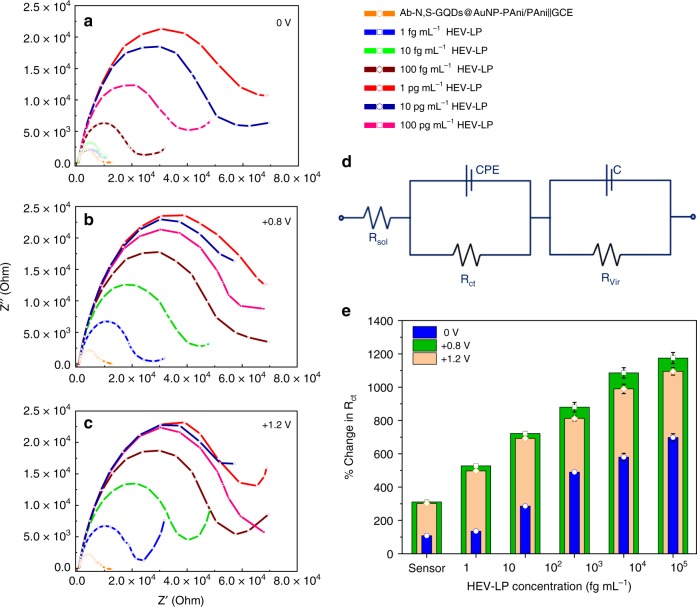
Electrochemical impedimetric performances over electric pulse on the sensor electrode with different virus concentration: EIS of Ab-N,S-GQDs@AuNP-PAni/PAni||GCE sensor electrode before and after incubation with 1 fg mL^−1^ to 100 pg mL^−1^ of HEV-LP with external pulses of **a** 0 V, **b** +0.8 V, and **c** +1.2 V. Circuit diagram of the proposed sensor (**d**) and comparative diagram of percentage change in *R*_ct_ values in three pulse conditions (**e**). Error bars represent the standard deviation of triple measurements

The Nyquist impedance plots of the Ab-N,S-GQDs@AuNP-PAni/PAni||GCE sensor electrode before and after virus loading are shown in Fig. 4a–c, where *Z*′ represents the real part and *Z*′′ the imaginary part of the complex impedance over the frequency range 100 kHz–100 mHz with an AC amplitude of 5 mV. The electrochemical impedance spectroscopy (EIS) responses of the sensor electrodes corresponding to the addition of different concentrations of HEV-LP in the range of 1 fg mL^−1^ to 100 pg mL^−1^ are shown in Fig. 4a‒c.

In the first case, shown in Fig. 4a, the Nyquist plot clearly shows that the change in impedance for relatively high concentrations of virus samples (100 fg mL^−1^ to 100 pg mL^−1^) are quite satisfactory compared with other impedimetric reports^41–45^. However, satisfactory information is not obtained for virus concentrations less than 100 fg mL^−1^. To achieve more sensitive detection, an external pulse was applied during the virus loading time, as depicted in the scheme in Fig. 1. For the electroporation-mediated process, surface permeability and expansion correlate strongly with the pulse intensity^46,47^. In this work, to determine the optimal conditions for virus sensing, the dependence of the loading efficiency of HEV on the electrical parameters of pulse intensities of 0, +0.8 and +1.2 V was investigated through the impedance. At both +0.8 and +1.2 V, the sensitivity is greatly enhanced in the low analyte concentration range (Fig. 4b, c) compared to that without pulsed conditions. However, with the higher pulse of +1.2 V, the sensitivity is slightly disturbed at high concentrations of virus (10 pg to 100 pg mL^−1^). This disturbance may be due to possible damage to the polyamine and GQD-based sensor matrix at this high-applied potential^48^.

To examine the individual contributions of the sensor electrodes, these Nyquist plots are fitted with several possible equivalent circuit diagrams over frequencies ranging from 100 kHz to 1 Hz, and the best fitted diagram is depicted in Fig. 4d. The electronic and ionic currents around the electrode surface are governed by several factors, viz., the bulk solution resistance (*R*_sol_), the double layer capacitance (*C*) formed by the ions at the vicinity of the electrode, the charge transfer resistance (*R*_ct_) that represents the current flow due to redox reactions at the electrode electrolyte interface, a constant phase element and the possible resistance of the attached virus layer with the electrode (*R*_Vir_). The Nyquist graphs and fitted values show that the most significant changes occur in the charge transfer resistance (*R*_ct_) and the virus resistance (*R*_Vir_) values. The *R*_ct_ values of these three systems are compared in Fig. 4e, and it can be concluded that the +0.8 V external pulse during the virus loading process is the best suited condition for a wide range of virus concentrations.

To examine the individual role of each component in GCE||PAni/AuNP-PAni/N,S-GQD-Ab electrode, step by step control experiments have been carried out at identical condition in presence of 10 pg mL^−1^ HEV-LP (Supplementary Fig. 7a–d). Without antibody loading, the GCE||PAni/AuNP-PAni/N,S-GQD electrode shows excellent electroactivity but fails to recognize the virus (Supplementary Fig. 7b) compared with the antibody-loaded sensor electrode (Supplementary Fig. 7a). A small increase of impedance has been noticed which may be due to the physical adsorption of the negatively charged viruses on the polyaniline chain (Supplementary Fig. 7b). Without loading of Ab-N,S-GQDs, the GCE||PAni/AuNP-PAni electrode shows similar nature in Nyquist plot with less conductivity (Supplementary Fig. 7c). The additional resistance comes due to the absence of the highly conducting N,S-GQD on the electrode surface (Supplementary Fig. 7d). In addition, the bare AuNPs were also drop-casted in the PDDA-coated GCE electrode to examine any interaction of AuNP with HEV-LP. Similar impedance curve of GCE||AuNP after HEV-LP addition proves the inertness of AuNP towards viruses. Its only function is to attach the sulfur-doped GQDs on the PAni chain via gold thiol bonding.

### Calibration of HEV detection on external pulse of +0.8 V

**Fig. 5:**
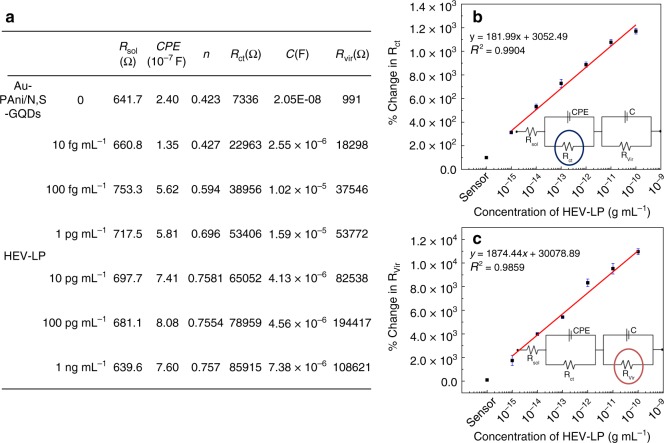
Electrochemical parameters of the sensor electrode obtained from impedimetric circuit diagram: **a** before and after immobilization of each concentration of HEV-LP; calibration lines obtained from **b** *R*_ct_ vs. HEV-LP concentration, and **c** *R*_Vir_ vs. HEV-LP concentration. Circles in the insets in **b** and **c** denote the measurable component of the circuit diagram. Error bars represent the standard deviation of triple measurements

The initial value of the charge transfer resistance may be primarily contributed by the electron transfer between the Ab-N,S-GQDs@AuNP-PAni/PAni||GCE sensor electrode and electrolyte. After HEV-LP loading, a large number of nonconducting virus molecules covered the conducting surface of GQDs and AuNP-PAni, resulting in an increase in the charge transfer resistance. As shown in Fig. 5a, the solution resistance does not change significantly since neither the charge transfer process nor the ion movement process in the double layer alters the mobile ion concentration in the solution. From the circuit diagram model, it can be concluded that the nature of the ionic movement near the sensor electrode with electrolyte is mainly influenced by a constant phase element^21^. After virus loading, the double layer capacitance usually increases, probably due to a decrease in the Debye length or increase in the potential of the zero charge value^48^. The value of *n* is an indicator of surface roughness, which does not change significantly since the HEV-LP and the antibody layers are smooth compared to the roughness of the N,S-GQDs@AuNP-PAni nanocomposites. The percentage changes in the signal difference between the *R*_ct_ values of the virus-loaded electrode and the bare Ab-N,S-GQDs@AuNP-PAni/PAni||GCE sensor electrode is adopted as the measurement signal to obtain the calibration lines. The calibration plot displays a good linear relationship between *R*_ct_ and the logarithmic values of HEV-LP concentration in the range of 1 fg mL^−1^ to 100 pg mL^−1^, as shown in Fig. 5b. In addition, the limit of detection (LOD), determined by 7*σL* (where *σ* is the standard deviation of the lowest signal and *L* is the lowest concentration used)^38^, is found to be 0.8 fg mL^−1,^ which is exceptionally low due to the pulse induction. Similarly, *R*_Vir_ also shows linearity in the same concentration range (Fig. 5c). As the sensor electrode does not make any real contribution to *R*_Vir,_ the bare electrode value is nearly negligible compared to those of other virus-loaded electrodes. The linearity trend found in *R*_Vir_ vs. HEV-LP is not as accurate as *R*_ct_, but it can help to cross verify the values found in the first linear calibration of *R*_ct_ vs. HEV-LP. The two calibration lines obtained from the fitted values of the impedimetric results of virus-loaded sensor electrodes can be used for any unknown sample analysis with improved accuracy.

### Selectivity and stability of the biosensor

**Fig. 6:**
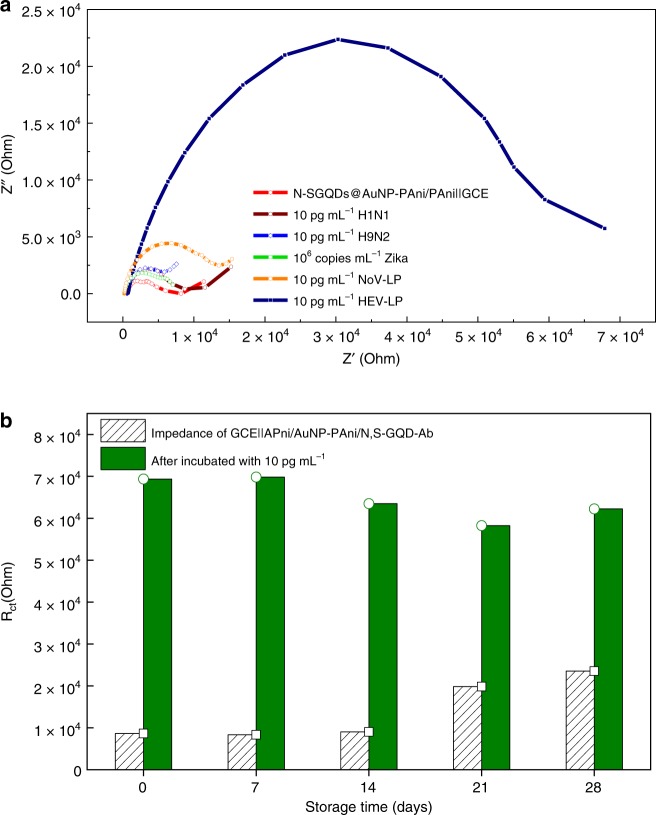
Selectivity and stability of the proposed sensor. **a** Influenza virus A (H1N1 and H9N2), zika virus and NoV-LP were used for the selectivity test. **b** Stability of the GCE||PAni/AuNP-PAni/N,S-GQD-Ab electrode with (green bars) and without (shaded bars) 10 pg mL^−1^ HEV-LP incubation over 4-week period

As the main interaction between the analyte virus molecule of virus and the sensor electrode is governed by the monoclonal antibody-conjugated nanocomposites, it is clear that the biosensor should possess high specificity. To investigate the selectivity of the biosensor, the sensor electrode has been tested with other samples of influenza virus A (H1N1 and H3N9) and norovirus-like particle (NoV-LP) of 10 pg mL^−1^ and zika virus of 10^6^ copies mL^−1^ (Fig. 6a). As shown in the Nyquist plot, the biosensor responses to the other viruses are significantly lower due to the nonspecific interaction with the antibody, which makes the sensor specific for the target analyte. The fitted values of these electrodes are also presented in Supplementary Table 1: the *R*_ct_ and *R*_Vir_ of these viruses are 8748, 8091, 8045, and 15,345 Ω and 1902, 1144, 2305, and 16,031 Ω, respectively, which are almost negligible with respect to the resistance found for the HEV-LP-loaded electrode (65,052 and 82,538 Ω).

In order to examine the stability of the sensor, the HEV-LP detection was performed over 1-month period. As shown in Fig. 6b, the prepared electrode, preserved at 4 °C, can be able to apply within 2 weeks from its preparation without compromising its initial performance.

### Detection of HEV-LPs in serum and HEVs in cell supernatants

**Fig. 7:**
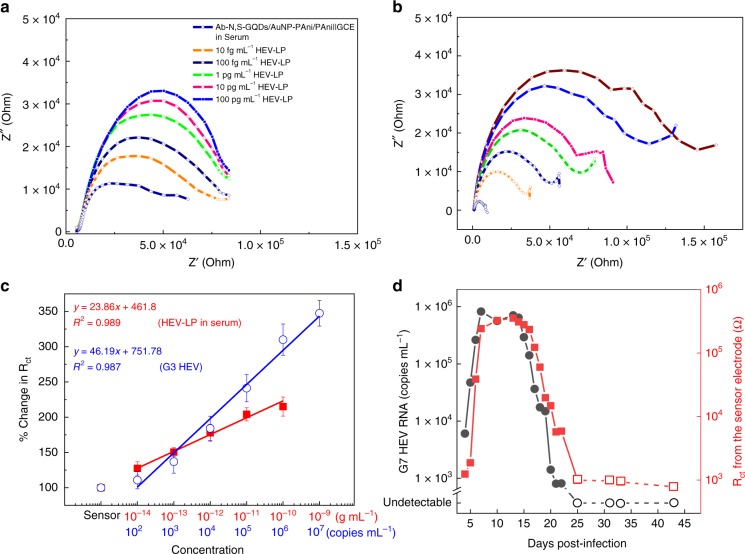
Sensor performances in serum and different HEV analytes. Detection of HEV-LP in human serum (**a**) and G3 HEV in cell culture supernatants (**b**). **c** *R*_ct_ vs. HEV for both cases of HEV-LP (closed squares) in serum and G3 HEV (open circles) in cell culture supernatants. Error bars represent the standard deviation of triple measurements. **d** Comparison of the sensitivity between the impedimetric sensor (closed squares) and RT-qPCR (closed circles) by detection of G7 HEV in fecal specimens. The fecal specimens were collected at 4–43 pdi from an experimentally G7 HEV-infected cynomolgus macaque monkey. Open circles represent samples in which the viral RNAs are undetectable by RT-qPCR

To validate the feasibility of the proposed sensing methodology, the detection of different concentrations of HEV-LP in the serum prior to naive HEV analysis is extremely important. The impedimetric signal responses of the biosensor were recorded in blank 50% serum first and taken as the bare electrode value for all further analyses. The presence of a very large amount of unspecified proteins in the serum matrix leads to some nonspecific interaction with the sensor electrode, resulting in a relatively large impedance value of 8339 Ω for the bare sensor electrode (Fig. 7a). In addition, the pattern of all impedimetric curves changes slightly from their previously measured experiments in buffer due to the higher double layer capacitance formed on the surface of the sensor electrode because of the interference of serum. However, the fitted values of the two measuring parameters *R*_ct_ and *R*_Vir_ show the same trends (Supplementary Table 2) as before.

To examine whether the proposed method could be used for detection of native HEV, the impedimetric responses are tested in four samples of G1, G3, G7, and ferret HEV collected from cell culture supernatants. The biosensor responses show a similar trend of increasing impedance with respect to the increasing concentration of G3 HEV (Fig. 7b), as well as G1, G7, and ferret HEV (Supplementary Fig. 8). In the case of serum samples, the calibration lines for *R*_ct_ vs. HEV-LP concentration in the range of 10 fg mL^−^^1^ to 100 pg mL^−1^ are plotted in Fig. 7c. The 1 fg mL^−1^ did not respond well due to the initial high resistance of the serum-loaded electrode. However, the linearity found with a correlation coefficient of 0.989 suggests that though the proposed biosensor is affected by biological interferences, its applicability is still appreciable for low-virus concentrations in the range of 10 fg mL^−1^ to 100 pg mL^−1^ and that can be further applied for determination of clinical samples. In case of HEVs from culture supernatants, the linearity found in the wide concentration range of 10^2^–10^7^ copies mL^−1^ of these HEVs with a detection limit of 96.7 copies mL^−1^ (determined by 7*σL*). As the antibody used here is rabbit anti-G3 HEV-LP, it is simple to obtain a highly optimized Nyquist impedance plot for the G3 sample (Fig. 7b). The fitted values of *R*_ct_ and *R*_Vir_ increased as expected (Supplementary Table 2). The calibration lines for *R*_ct_ found from G3 HEV impedance are plotted against the concentration, which follows the same pattern as in the buffer samples (Fig. 7c). Due to presence of high amount of interferences, the correlation coefficients are found 0.987 for the *R*_ct_ which was not as good as buffer samples are, however, quite satisfactory in for application. The *R*_Vir_ vs. virus concentration calibration line for HEV-LP in serum and G3 HEV in cell culture supernatants are given in Supplementary Fig. 9.

### HEV detection in fecal specimens

To further examine the availability of the method developed in this study, a series of fecal specimens containing 19 samples collected at 4–43 days post infection (dpi) from experimental G7 HEV-infected cynomolgus monkey were tested^49^. The HEV RNA was detectable in fecal specimens from 4 to 22 dpi by RT-qPCR. Since the samples collected at 25, 31, 33, and 43 dpi were undetectable in RT-qPCR, these samples are treated as negative controls. The cutoff value was calculated as 1841 Ω for the proposed detection method on the basis of two times of mean *R*_ct_ values of negative controls. As shown in Fig. 7d, the G7 HEV was detected in monkey fecal specimens from 5 to 22 dpi, whereas only one sample, collected at 4 dpi was near to the *R*_ct_ cutoff value. The correlation between the copy numbers by RT-qPCR and *R*_ct_ values by the sensor was showed in Supplementary Fig. 10. These results suggested that the sensitivity of our proposed method is comparable to that of RT-qPCR.

## Discussion

The central theme of this work is schematically presented in Fig. 1, which illustrates a modified pulse-induced method of electrochemical impedimetric sensing using N,S-GQDs@AuNP-PAni nanocomposites as the electrode matrix. Although impedimetric analysis has already been well explored in the past decade, introducing the electrical pulse to the sensing process provides a strategy that allows virus sensing methodologies to achieve at least tenfold lower detection limits compared with other conventional methods. In summary, we have realized from this current work that the pulse-induced mechanism for impedimetric sensing can provide a more sensitive tool for the detection of virus particles in real applications. To design the electrochemical biosensor, Ab-N,S-GQDs@AuNP-PAni nanocomposites were drop-cast on a finely PAni-coated GCE. The AuNP-PAni enhances the electron transfer process and supports a large surface area on which to conjugate monoclonal antibody-conjugated N,S-GQDs, which improve the electrochemical response and provide enhanced active sites for the target HEV. To obtain more sensitive detection in lower concentration of virus samples, the sensor was applied different external pulses were applied to the sensor during the virus incubation time, which was optimized to achieve the best results at +0.8 V. Under this optimal condition, the proposed biosensor demonstrates its ability to detect HEV-LP in a wide concentration range from 1 fg mL^−1^ to 100 pg mL^−1^ with a low-detection limit of 0.8 fg mL^−1^. According to our hypothesis, the electric pulse enhances the sensitivity of the sensor electrode which can be explained by three possible reasons. First, the expansion of polyaniline chain length on the working electrode during the electric pulse. Polyaniline is already well-known for its actuator properties which can be regulated from the electric pulse^50,51^. From its earlier studies, it can be shown that in presence of positive charge, the length of polyaniline chain can be increased which again returns back to its pristine structure while the electric pulse is off^52^. This expansion during redox switching can be occurred due to the changes in bond length and conformation in the polymer backbone^53^. In this work, as the AuNP which is attached with the Ab-N,S,GQDs is embedded on the surface of the polyaniline chain; when the chain has expanded, the antibody that was conjugated to N,S,GQDs has also been exposed more toward the sensing solution, resulting higher possibility for accumulation of larger amount of viruses on the electrode surface. Second, the external pulse would expose the surface of viruses, which facilitates to increase the probability of binding virus to the antibody-conjugated N,S-GQDs. In a study on virus-based piezoelectric energy generation, a virus-coated film generates electricity when an external compression has been given^54^. In contrast to that report, here, we assumed that the given electric pulse can expand the virus surfaces, in very small scale offering more exposed surface markers towards antibody^55^. DLS data of the bare HEV-LPs before and just after the electric pulse show a small increase in the size of HEV-LPs (Supplementary Fig. 11), supporting our assumption. Though the expansion of the polyaniline chain as well as virus surface are taken place in extremely small order, however, this can be enough to make the impact on the short domain of the working electrode surface as well as the few nanometer-sized virus particles. Finally, the virus surface is strongly negatively charged with zeta potential of 9.8 mV in the working pH of 6.8 as recorded by the zeta potential measurement (Supplementary Fig. 12). In contrast, the emeraldine formation of the polyaniline chain also contain positively charged backbone48, which can enhance its positively charged environment during the +0.8 V of electrical pulse. Therefore, the negatively charged virus particle always have higher tendency toward positively charged polyaniline backbone, resulting higher virus accumulation.

The applicability of the proposed biosensor was also tested successfully in human serum and G1, G3, G7, and ferret HEVs in cell culture supernatants. Linearity was achieved in a wide concentration range of 10^2^–10^7^ copies mL^−1^ of these HEVs. Analysis of the research trends regarding virus detection reveals that most studies were conducted on the basis of DNA or RNA analysis from the extracted virus samples. In addition, secondary targets of induced antibodies or proteins in infected targets are also very popular as target sensor analytes. However, direct virus detection is clearly more reliable than DNA or antibody detection because it reduces the chance of false positive or negative responses, which are unfortunately difficult to establish. Table 1 shows that most virus detection protocols are mainly focused on DNA or RNA analysis of different viruses, whereas few of them address real samples. Among the few reports on clinically isolated virus detection, Takemura et al.^6^ reported a fluorometric analysis based on localized surface plasmon resonance where the detection limit of influenza virus was found to be on the order of 10^−10^ g mL^−1^. In another report, Zeng et al. established an LOD of 8.3 × 10^−9^ g mL^−1^ for HBV detection^56^. However, in this current study, we achieved much higher sensitivity, on the order of femtograms, due to the application of a sensitive impedimetric method with the introduction of an electrical pulse. Due to the usage of antibody–antigen interaction, the sensor shows extremely good selectivity in presence of other viruses, serum and even culture medium. As it is very difficult to obtain clinical specimens immediately after HEV infection, experimental infection in monkeys is positioned as a model for reproducing human infection. In that case, the developed method can detect the G7 HEV in experimental G7 HEV-infected monkey’s fecal specimens at 5–22 dpi, which strongly recommended that the sensor can offer a useful platform for virus detection in near future.

**Table 1:** Comparison with recently reported virus detection methods

Analyte	Linear range	LOD	References
Influenza virus (Fluorometric)	1–10 × 10^−11^ g mL^−1^	3 × 10^−10^ g mL^−1^	^6^
Norovirus RNA (Colorimetric)	10^2^–10^6^ copies mL^−1^	13.2 copies mL^−1^	^2^
Influenza A virus (Colorimetric)	5 × 10^−15^–5 × 10^−6^ g mL^−1^	44.2 × 10^−15^ g mL^−1^	^61^
Influenza A virus (Fluorometric)	50–1.0 × 10^4^ PFU mL^−1^	50 PFU mL^−1^	^62^
Dengue RNA (Fluorometric)	5–500 × 10^−9^ g mL^−1^	5.2 × 10^–9^ g mL^−1^	^56^
HBV (Fluorometric)	>264 × 10^−9^ g mL^−1^	8.3 × 10^–9^ g mL^−1^	^63^
Norovirus RNA (Microfluidic)	1–3.5 × 10^−9^ M	1 × 10^−11^ M	^64^
Norovirus RNA (Fluorometric)	2–18 copies mL^−1^	1.2 copies mL^−1^	^65^
HBV (Fluorometric)	0.01–1 IU mL^−1^	0.4 IU mL^−1^	^66^
HEV RNA (RT-qPCR)	10^3^–10^6^ IU mL^−^^1^	2.1 × 10^4^ IU mL^−1^	^67^
HEV RNA (RT-qPCR)	8.75 × 10^3^–8.75 × 10^4^ copies mL^−1^	8.75 × 10^3^ copies mL^−1^	^11^
HEV RNA (RT-qPCR)	10–10^9^ copies mL^−1^	10 copies mL^−1^	^68^
HEV RNA (RT-qPCR)	2.01 × 10^3^–1.71 × 10^5^ GC g^−1^	10 GC g^−^^1^	^57, 69, 70^
HEV-LP	10^−12^ –10^−15^ g mL^−1^	8 × 10^−14^ g mL^−1^	This work
HEV (pulse impedance)	10^2^–10^7^ RNA copies mL^−1^	96.7 RNA copies mL^−1^	This work

## Methods

### Reagents

Sulfuric acid, sodium acetate, and acetone were purchased from Wako Pure Chemical Ind. Ltd. (Osaka, Japan). Toluene, aniline, thiourea, citric acid, 1-ethyl-3-(3-dimethylaminopropyl) carbodiimide hydrochloride (EDC), N-hydroxysuccinimide (NHS), HAuCl_4_, and human serum were purchased from Sigma-Aldrich (St. Louis, USA). All aqueous solutions were prepared using high-purity deionized (DI) water (>18 MΩ cm^−1^). Tetramethylbenzidine (TMBZ) was purchased from Dojindo (Kumamoto, Japan).

### Synthesis of N, S-GQDs

N,S-GQDs were prepared by a standard hydrothermal method with thiourea to citric acid^28^. In brief, 0.23 g citric acid and 0.23 g thiourea were dissolved into 5 mL of DI water and then transferred into a 20-mL Teflon lined stainless steel autoclave tube. Then, the solution was heated to 160 °C for 4 h to obtain a brown suspension of N,S-GQDs. It was then added to an ethanol solution and centrifuged at 5000 × *g* for 5 min to remove the excess reagents. To obtain uniform size and further purification, the as-prepared N,S-GQDs were dialyzed with a 1 kDa dialysis bag for 8 h.

### Synthesis of AuNP-PAni nanocomposites

Au-PAni was synthesized using the interfacial polymerization method^31^. Three millimolar HAuCl_4_ in 0.1 M HCl aqueous solution was slowly poured into 0.5 M aniline monomer in toluene as an organic phase to initiate the interfacial polymerization process. Polyaniline nanofibers slowly formed in the aqueous phase, and the solution color became dark green within several minutes. AuNPs were also synthesized and combined at the same time within the polyaniline nanofiber in aqueous phase during the polymerization process. The synthesized solution was centrifuged at 5500 × *g* at room temperature and re-dispersed using ultrapure water for purification. This purification process was performed three times.

### Antibody conjugation of N,S-GQD

N,S-GQDs were easily bonded with antibody using EDC/NHS covalent chemistry. In brief, 0.1 M EDC was mixed with 5.1 µg of antibody solution (in phosphate-buffer saline (PBS)), and EDC reacted with the carboxyl group of the antibody to create an active-ester intermediate within 30 min of stirring at room temperature. Then, 0.1 M NHS and 1 mL of N,S-GQD were added to enable an amine reaction between an amino group and the surface of GQD and stirred continuously at 7 °C for 16 h. The reaction solution was dialyzed using a 1 kDa dialysis bag to remove unreacted EDC and NHS. Finally, the stock solution of antibody-conjugated N,S-GQD (Ab-N,S-GQD) in 0.1 M phosphate-buffer saline (PBS, pH 7.4) was stored at 4 °C.

### Characterization of nanocomposites

HRTEM images of the nanocomposites were taken by TEM operating at 200 kV (JEM-2100F, JEOL, Tokyo, Japan). In the case of purified HEV-LP, samples were placed on a carbon-coated grid for 45 s, rinsed with distilled water, stained with a 2% uranyl acetate solution and examined with a transmission electron microscope (TEM-1400, JEOL) operating at 80 kV. Electrochemical characterization of CV and EIS were carried out on an SP-150 (BioLogic.Inc., Tokyo, Japan) in a conventional three-electrode cell with an Au disk electrode (4 mm in diameter), platinum wire and saturated Ag/AgCl as the working, auxiliary, and reference electrodes, respectively (EC frontier, Tokyo, Japan). The pulse was generated during the virus incubation time with two Pt wires as the electrodes. Differences in nanoparticle complex formation between Ab-N,S-GQD@AuNP-PAni and AuNP-PAni were accounted for through X-ray photoelectron spectroscopy (XPS, ESCA1600 system, ULVAC-PHI Inc., Tokyo, Japan) using an Al Kα X-ray source (1486.6 eV) and a hemispherical electron analyzer. Powder X-ray diffraction analysis was carried out using a RINT ULTIMA XRD (Rigaku Co., Tokyo, Japan) with a Ni filter and a Cu-Kα source. Data were collected over 2*θ* = 5–90° at a scan rate of 0.01°/step and 10 s/point. Zeta potential and dynamic light scattering (DLS) measurements were performed using a Zetasizer Nano series (Malvern Inst. Ltd., Malvern, UK). TGA was carried out using a differential thermal balance (Thermo plus EV02, Rigaku, Tokyo, Japan).

### Construction of a recombinant baculovirus

A genotype 1 (G1) HEV strain (GenBank accession no.: DQ079624) was isolated from a patient with acute hepatitis E in Myanmar in 1986. DNA fragments with deletions of the N-terminal 111 amino acids encoded by ORF2 were amplified by PCR with two primers, HEV-D13 (5′-AA*GGATCC*ATGGCGGTCGCTCCAGCCCATGACACCCCGCCAGT-3′) and HEV-U14 (5′-GG*TCTAGA*CTATAACTCCCGAGTTTTACCCACCTTCATCTT-3′). The PCR product contained the *Bam*HI site before the start codon and the *Xba*I site after the stop codon. Then, the PCR product was purified using a QIAquick Gel Extraction Kit (Qiagen, Hilden, Germany) and cloned into TA cloning vector pCR2.1 (Invitrogen, Carlsbad, CA, USA), digested with *Bam*HI and *Xba*I, and ligated with a baculovirus transfer vector, pVL1393 (Pharmingen, San Diego, CA, USA), to yield plasmid pVL5480/7126. To produce the recombinant baculovirus, the transfer plasmid pVL5480/7126 was mixed with baculoGold (Pharmingen) and lipofectin (GIBCO-BRL, Gaithersburg, MD, USA) and transfected into insect cells Sf9 (Riken Cell Bank, Tsukuba, Japan). The cells were incubated at 26.5 °C in TC-100 medium (GIBCO-BRL) supplemented with 10% FBS and 0.26% tryptose phosphate broth (Difco Laboratories, Sparks, MD, USA). The recombinant viruses were plaque-purified three times in Sf9 cells and designated as Ac5480/7126^57^.

### Expression and purification of HEV-LPs

Cells from the insect cell line from *Trichoplusia ni*, BTL-Tn 5B1–4 (Tn5), were transfected with recombinant baculovirus Ac5480/7126 at a multiplicity of infection of 10 and cultured in EX-CELL 405 medium (JRH Biosciences, Lenexa, KS, USA) at 26.5 °C. The Ac5480/7126-infected Tn5 cells were harvested at 7 dpi. The intact cells, cell debris, and progeny baculoviruses were removed by centrifugation at 10,000 × *g* for 60 min. The supernatant was then spun at 100,000 × *g* for 3 h in a Beckman SW32Ti rotor, and the resulting pellet was resuspended in EX-CELL 405 medium at 4 °C overnight. For the CsCl gradient centrifugation, 4.5 mL of each sample was mixed with 2.1 g of CsCl and then centrifuged at 100,000 × *g* for 24 h at 10 °C in a Beckman SW55Ti rotor. The gradient was fractionated into 250-μL aliquots, and each fraction was weighed to estimate the buoyant density and isopycnic point. To remove the CsCl, each fraction was diluted with EX-CELL 405 medium and centrifuged for 2 h at 100,000 × *g* in a Beckman TLA55 rotor^57^.

### Rabbit anti-G3 HEV IgG antibody generation

Japanese white rabbits were immunized with G3 HEV-LPs. Immunization was performed by the percutaneous injection of purified G3 HEV-LPs at a dose of 500 μg per rabbit, and booster injections were carried out 4 and 6 weeks after the first injection with half doses of G3 HEV-LPs. All of the injections, including booster injections, were carried out without adjuvant. Blood was taken from the immunized animals 3 weeks after the last injection, and the anti-HEV-LPs IgG was purified by protein G column^58^.

### Infectious HEVs and HEV RNA measurement

G1, G3, G7, and ferret HEVs were produced by cell culture with a human hepatocarcinoma cell line, PLC/PRF/5 (JCRB0406, the Health Science Research Resources Bank, Osaka, Japan)^49,59^. The cells were grown in Dulbecco’s modified Eagle’s medium (DMEM) supplemented with 10% (v/v) heat-inactivated fetal bovine serum (FBS) at 37 °C in a humidified 5% CO_2_ atmosphere. For virus inoculation, a total of 1 ml of the 10% stool suspension sample was inoculated onto PLC/PRF/5 cells, and incubated at 36 °C with 10 ml maintenance medium consisting of medium 199 (Invitrogen, Carlsbad, CA) containing 2% (v/v) heat-inactivated FBS and 10 mM MgCl_2_. The culture medium was replaced every 4 days and the HEVs in cell culture supernatants were used in this study. The RNA copy numbers of G1, G3, G7, and ferret HEVs were 3.7 × 10^8^, 1.8 × 10^8^, 5.0 × 10^8^, and 4.8 × 10^8^ copies mL^−1^, respectively. Detailed information on the RT-qPCR is given in the supporting information in Supplementary method 1–3.

As the nonhuman primates including cynomolgus macaques are widely used in animal models for the study of HEV infection and its pathogenesis and vaccine trials, the sensor performance has been tested in that. A series of fecal specimens containing 19 HEV samples was collected from 4 to 43 dpi from a G7 HEV-infected cynomolgus monkey^58^. The fecal specimens were diluted with 10 mM in PBS to prepare a 10% (w/v) suspension. Then the suspension was shaken at 4 °C for 1 h, clarified by centrifugation at 10,000 × *g* for 30 min, passed through a 0.45 µm membrane filter (Millipore, Bedford, MA), and stored at −80 °C until use. All of the HEV samples were inactivated by incubation at 70 °C for 20 min before perusing any sensing operation.

For selectivity test, zika virus was kindly provided by Professor K. Morita of Institute of Tropical Medicine, Nagasaki University. NoV-LPs were prepared according to our previous protocol^60^. Influenza virus A (H1N1) (New Caledonia/20/99) and (H9N2) (Hong-Kong/1073/99) were purchased from Prospec-Tany TechnoGene Ltd. (Rehovot, Israel).

### Preparation of the sensor electrode

For this purpose, 0.5 M sulfuric acid and 0.1 M aniline monomer were mixed in Millipore water for polymerization on a glassy carbon electrode (GCE). The GCE was set in 3 mL of aniline in 0.1 M sulfuric acid and deposited by cyclic voltammetry (CV) in a three-electrode system. The CV was recorded at a scan rate of 20 mV s^−1^ in a potential range of 0–1 V for 10 cycles. Finally, 10 µL of Ab-N,S-GQD@AuNP-PAni solution was drop-cast on the polymerized GCE||PAni.

### Sensing of HEV-LPs or HEV

HEV-LPs or HEV solution was dropped on the Ab-GQD-AuNP-PAni-coated electrode and incubated for 5 min with Ab-GQDs at room temperature. The electrode was washed by gentle dripping of ultrapure water, and the virus solution was removed. The electrode was measured by CV and in the potential EIS mode. The Ab-N,S-GQD@AuNP-PAni sensor was scanned with an amplitude of 5 mV from 100 kHz to 0.1 Hz. The HEV-LP solution was diluted with 0.1 M PBS (pH 7.4) from 10 fg mL^−1^ to 1 ng mL^−1^. In addition, HEV-LPs were diluted in 100% human serum to test the stability of this EIS sensor.

### Reporting summary

Further information on research design is available in the Nature Research Reporting Summary linked to this article.

## Supplementary information

Supplementary information

Reporting Summary

## Data Availability

All characterizations data of the sensor materials and all the electrochemical data are available and provided from the corresponding authors upon reasonable request.
