# Supplementary material for: Electrical pulse-induced electrochemical biosensor for hepatitis E virus detection
Source: Nat Commun. 2019 Aug 19;10:3737. doi: 10.1038/s41467-019-11644-5 (PMC6700141; doi:10.1038/s41467-019-11644-5)
Supplement: Supplementary file 1 — Supplementary information [file 41467_2019_11644_MOESM1_ESM.pdf]

Supplementary Information

Electrical pulse induced electrochemical biosensor for hepatitis E virus detection

Ankan Dutta Chowdhury[a,#], Kenshin Takemura[b,#], Tian-Cheng Li[c], Tetsuro Suzuki[d], Enoch Y. Park[*,a,b]

Supplementary method 1.

RT-qPCR for the detection of G1, G3 HEV RNA

To determine the copy numbers of HEV RNA, a TaqMan assay was performed with a 7500 FAST Real-Time PCR System (Applied Biosystems, Foster City, CA) using TaqMan Fast Virus 1-step Master Mix (Applied Biosystems).[1] RT-qPCR targeting a 170-bp region of ORF3 was carried out with a forward primer (5'-GGTGGTTTCTGGGGTGAC-3'), a reverse primer (5'-AGGGGTTGGTTGGATGAA-3'), and a probe (5'-FAM-TGATTCTCAGCCCTTCGC-TAMRA-3') under the following conditions: 5 min incubation at 50 °C, a 20 s incubation at 95 °C, and 40 cycles of 3 sec at 95°C and 30 s at 60°C. A 10-fold serial dilution of the full-length G3 HEV RNA (10^7 to 10^1 copies) was used as the standard to quantitate the copy numbers. Amplification data were collected and analyzed with Sequence Detector software ver. 1.3 (Applied Biosystems).

Supplementary method 2.

RT-qPCR for the detection of DcHEV

To determine the DcHEV RNA copy numbers, a TaqMan assay was performed with a 7500 FAST Real-Time PCR System (Applied Biosystems, Foster City, CA) using TaqMan Fast Virus 1-step Master Mix (Applied Biosystems).[2] The primers were 900 nmol L^{-1} forward primer 5'-CCATGGAGGCCCATCAGTT-3' (nt 38–56) and 900 nmol L^{-1} reverse primer 5'-TCGGGGCGAAAAACAAGCTG-3' (nt 199–218). The probe was 250 nmol L^{-1} 5'-FAM-CCAATTCCGCTTTGGCGAATGC-TAMRA-3' (nt 110–131). The conditions for the one-step RT-qPCR were: 5 min at 50°C, a 20-s incubation at 95°C, and 50 cycles of 3-s at 95°C and 30-s at 60°C. A 10-fold serial

dilution of capped DcHEV RNA (10^7 to 10^1 copies) was used as the standard for the quantitation of viral genome copy numbers. Amplification data were collected and analysed with Sequence Detector software version 1.3 (Applied Biosystems).

Supplementary method 3.

RT-qPCR for detection of ferret HEV

To determine the ferret HEV RNA copy numbers, a TaqMan assay was performed by using a 7500 FAST Real-Time PCR System (Applied Biosystems, Foster City, CA) with TaqMan Fast Virus 1-step Master Mix (Applied Biosystems, Foster City, CA).[3] The primers consisted of 900 nmol L^{-1} forward primer (5'-TGAGTGGTGTTTTTATGGCGA-3', nt 4006-4026) and 900 nmol L^{-1} reverse primer (5'-CAAACTCAGAAAAATCATTCTCAAAGAC-3', nt 4082-4109), and 250 nmol L^{-1} probe (5'-6FAM-GCCATGCCGCGGTTTGAGGCGGCTGT-TAMRA-3', nt 4034-4059). The condition for the one-step RT-qPCR was 15 min at 48°C, a 10-min incubation at 95°C, and 50 cycles of 15 s at 95°C and 1 min at 60°C. The capped *in vitro*-transcribed RNA of ferret HEV was used as the standard to calculate the copy number of the RNA molecule. A 10-fold serial dilution of the RNA standards (10^7 to 10^1 copies) was used for the quantitation of viral genome copy numbers. Amplification data were collected and analyzed with Sequence Detector software version 1.3 (Applied Biosystems). This RT-qPCR system, with a sensitivity of 10 copies, was used exclusively for ferret HEV.

Supplementary Table 1. Electrochemical parameters obtained from impedance analysis based upon the proposed equivalent circuit for the selectivity test with two influenza A (H1N1 and H9N2), zika and Noro viruses.

	Virus concentration	R_{sol} (Ω)	*CPE* (10^{-7} F)	*n*	R_{ct} (Ω)	*C* (F)	R_{vir} (Ω)
Au-PAni/N,S-GQDs	0	641.7	2.40	0.423	7336	2.05×10^{-8}	997
HEV-LP	10 pg mL^{-1}	697.7	7.41	0.7581	65052	4.13×10^{-6}	82538
H1N1	10 pg mL^{-1}	669.5	1.60	0.5484	8748	1.36×10^{-7}	1902
H9N2	10 pg mL^{-1}	687.8	1.64	0.5437	8091	1.31×10^{-7}	1144
Zika	10 pg mL^{-1}	697.2	1.42	0.6936	8045	2.14×10^{-7}	2305
NoV-LP	10 pg mL^{-1}	721.4	1.66	0.7273	15345	5.24×10^{-7}	16031

Supplementary Table 2. Electrochemical parameters from equivalent circuit for the detection of HEV-LP in human serum and G3 HEV in cell culture supernatant.

Analyte	Quantity	R_{sol} (Ω)	CPE(10^{-7}F)	n	R_{ct} (Ω)	C (10^{-6} F)	R_{vir} (Ω)
Serum	0	742.1	1.23	0.924	8339	1.25	2253
HEV-LP	10 fg mL^{-1}	860.2	3.33	0.983	10623	1.55	6639
	100 fg mL^{-1}	872.7	3.22	0.987	12548	1.24	8863
	1 pg mL^{-1}	792.2	5.23	0.942	13836	1.77	12763
	10 pg mL^{-1}	813.2	5.34	0.934	17023	1.91	12643
	100 pg mL^{-1}	765.8	5.24	0.945	17934	2.63	13945
Cell solution	0	640.2	0.31	0.885	4018	2.43	673
HEV	10^2 copies mL^{-1}	682.6	0.62	0.911	6064	2.88	1623
	10^3 copies mL^{-1}	703.4	0.54	0.923	6069	2.78	1833
	10^4 copies mL^{-1}	666.2	0.87	0.921	9236	3.29	3510
	10^5 copies mL^{-1}	752.2	0.92	0.932	17363	3.67	4937
	10^6 copies mL^{-1}	687.7	0.85	0.958	20573	3.52	8723
	10^7 copies mL^{-1}	736.5	0.88	0.963	22425	3.75	10086

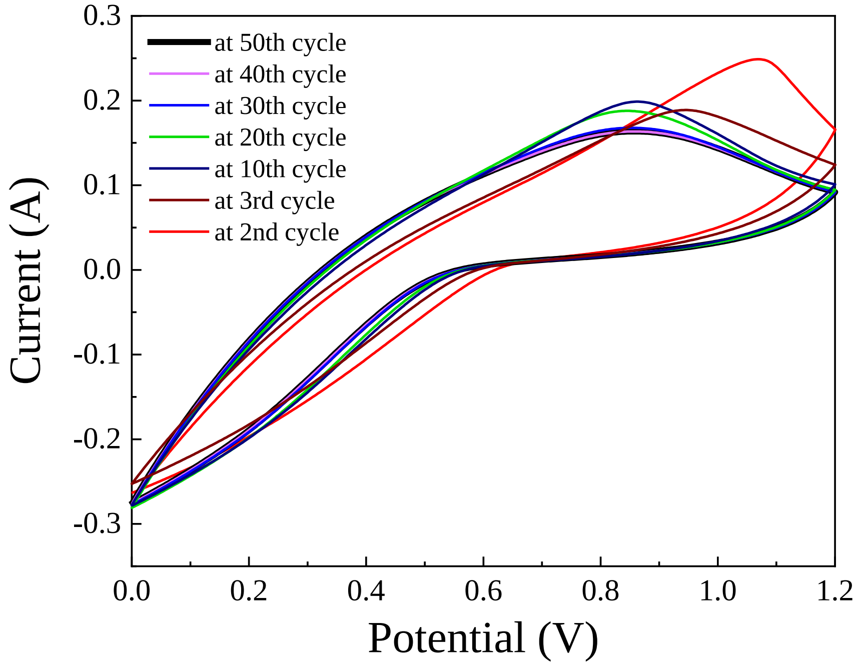

Supplementary Figure 1. Stability of the GCE||PAni/AuNP-PAni sensor electrode in cyclic voltammetry.

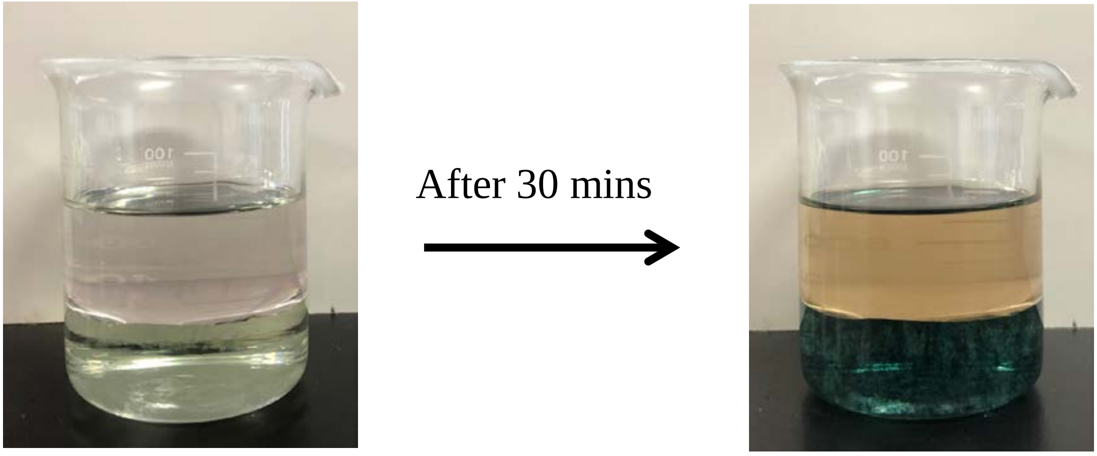

Supplementary Figure 2. Photo of synthesized Au-PAni nanocomposites: just after the formation of interfacial layer and after 30 minutes.

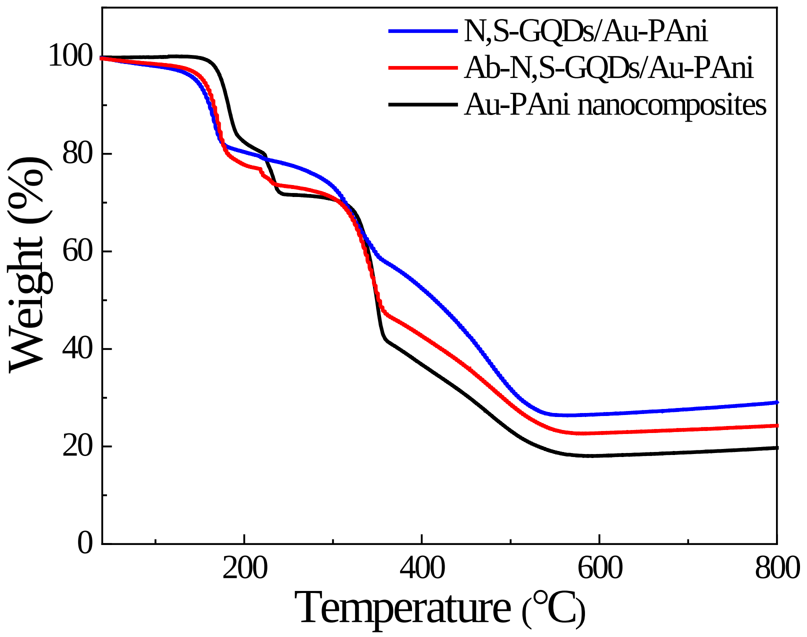

Supplementary Figure 3. TGA of AuNP-PAni, N,S-GQD/AuNP-PAni and Ab-N,S-GQD/AuNP-PAni nanocomposites. N,S-GQDs and antibody loading calculation from TGA are follows, % wt loss of bare AuNP-PAni: 81.1; increasing stability percentage for N,S-GQDs: 8.5; additional wt % loss for antibody addition: 2.4. It can be found from our earlier studies, GQDs have 86% of thermal stability in up to 800 ℃. From the calculation, it is found that approximately 0.3 μg mg^{-1} of N,S-GQDs and 0.4 μg mg^{-1} of antibody has been loaded on the AuNP-PAni nanocomposites.

Anti-HEV antibody specificity

100 ng mL^{-1} of HEV-LPs were added to a polystyrene 96-well plate (100 µL) and were incubated overnight at 4 °C. As a negative control, 100 µl of 2 % BSA was added to a separate well. Next, 100 µl of 5 % skim milk solution was added and applied as a blocking agent after washing 3 times with PBST (containing 1 mL of Tween in 999 mL of PBS buffer). After blocking, the 5 % skim milk was removed by washing 3 times with PBST. Anti-rabbit IgG-horseradish peroxidase was diluted to 1:4000 with 2 % BSA, and 100 µl of this solution was added to the well and was incubated at ambient temperature for 1 h. TMB (100 µl), a chromogenic substrate, was added to the well as a coloring reagent, and confirmation of this effect was indicated by a blue color. The reaction was stopped by adding 50 µL of 10 % H_2SO_4, which changed the color of the solution from blue to yellow. The absorbance of the solution was measured using a microplate reader at 450 nm with a reference filter of 655 nm.

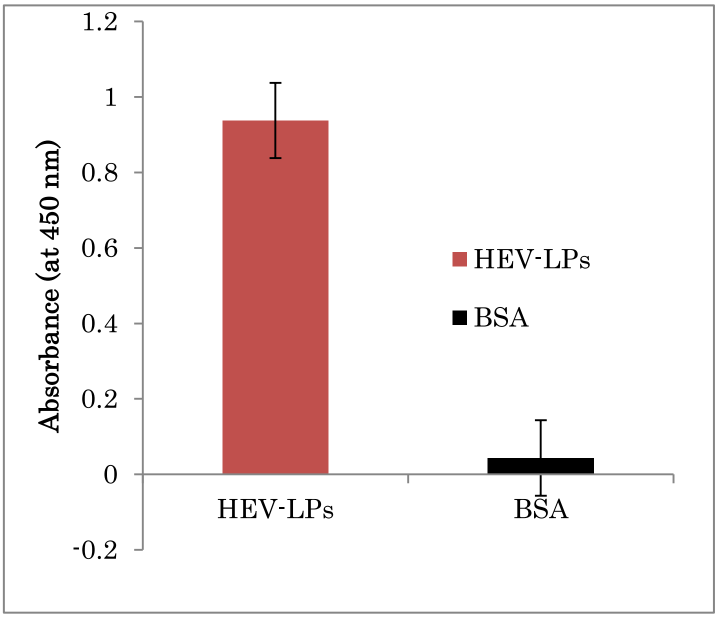

Supplementary Figure 4. Confirmation of antibody specificity for HEV-LPs by ELISA. Error bars represent the standard deviation of three samples.

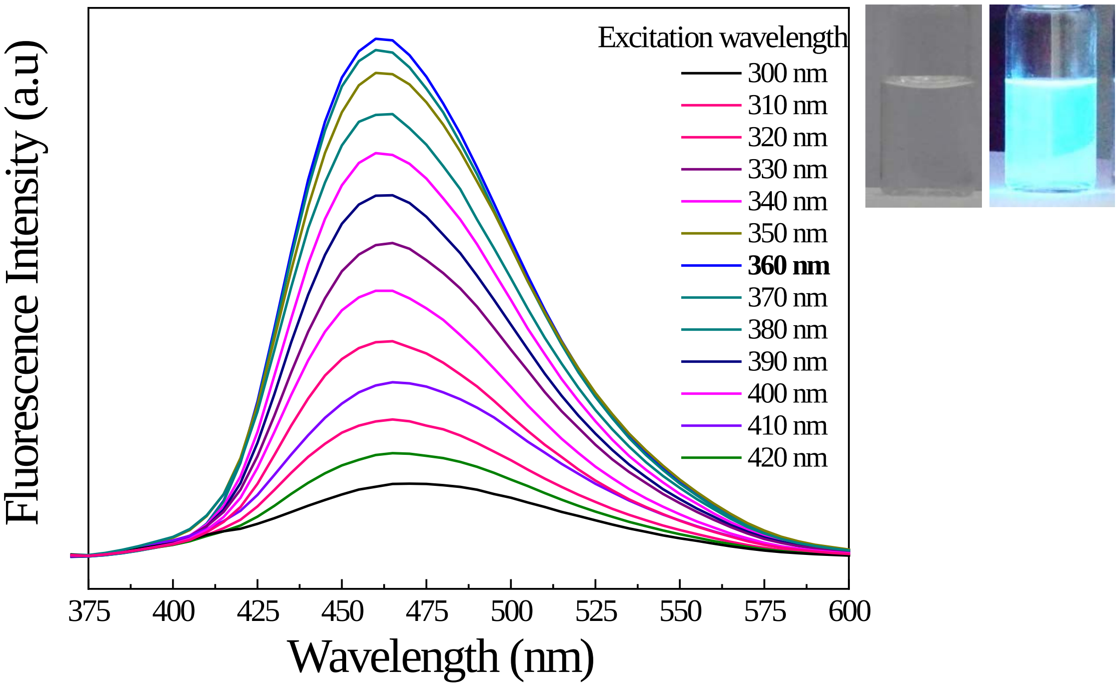

Supplementary Figure 5. Fluorescence spectra of N,S-GQDs: The excitation independent fluorescence and its image in 360 nm UV light confirms its synthesis.

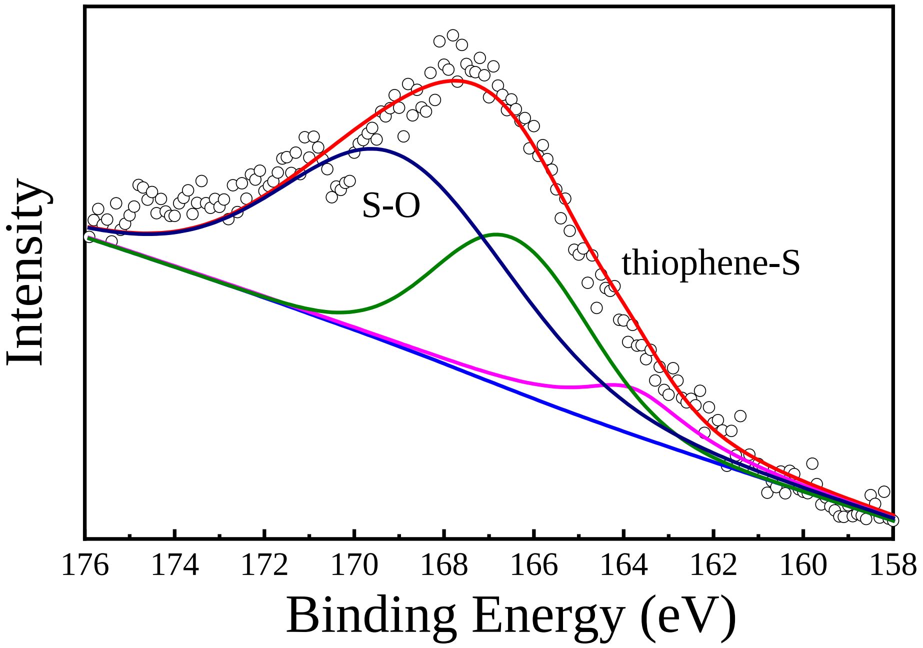

Supplementary Figure 6. Deconvoluted spectra of S 2p in N,S-GQDs@AuNP-PAni nanocomposites.

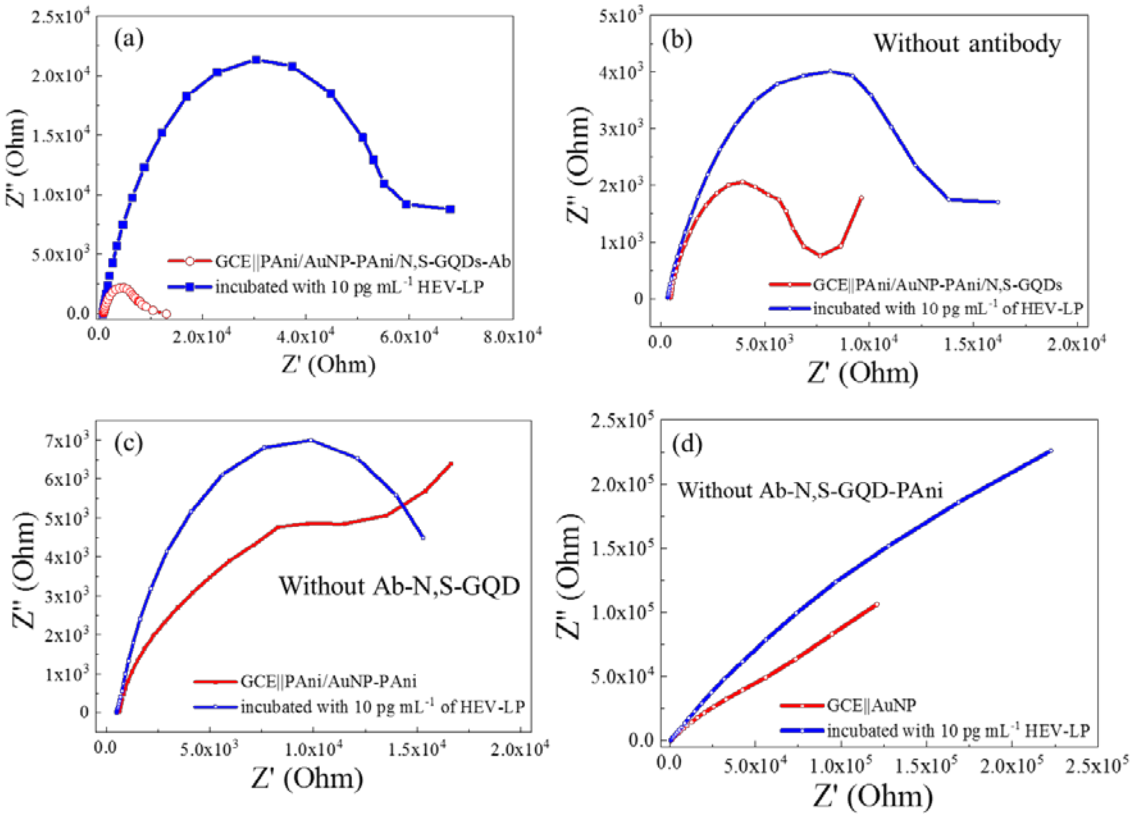

Supplementary Figure 7. Impedimetric curve of different sensing electrode before and after addition of 10 pg mL^{-1} HEV-LP; (a) GCE||PAni/AuNP-PAni/N,S-GQD-Ab, (b) GCE||PAni/AuNP-PAni/N,S-GQD without antibody loading, (c) GCE||PAni/AuNP-PAni and (d) GCE||AuNP.

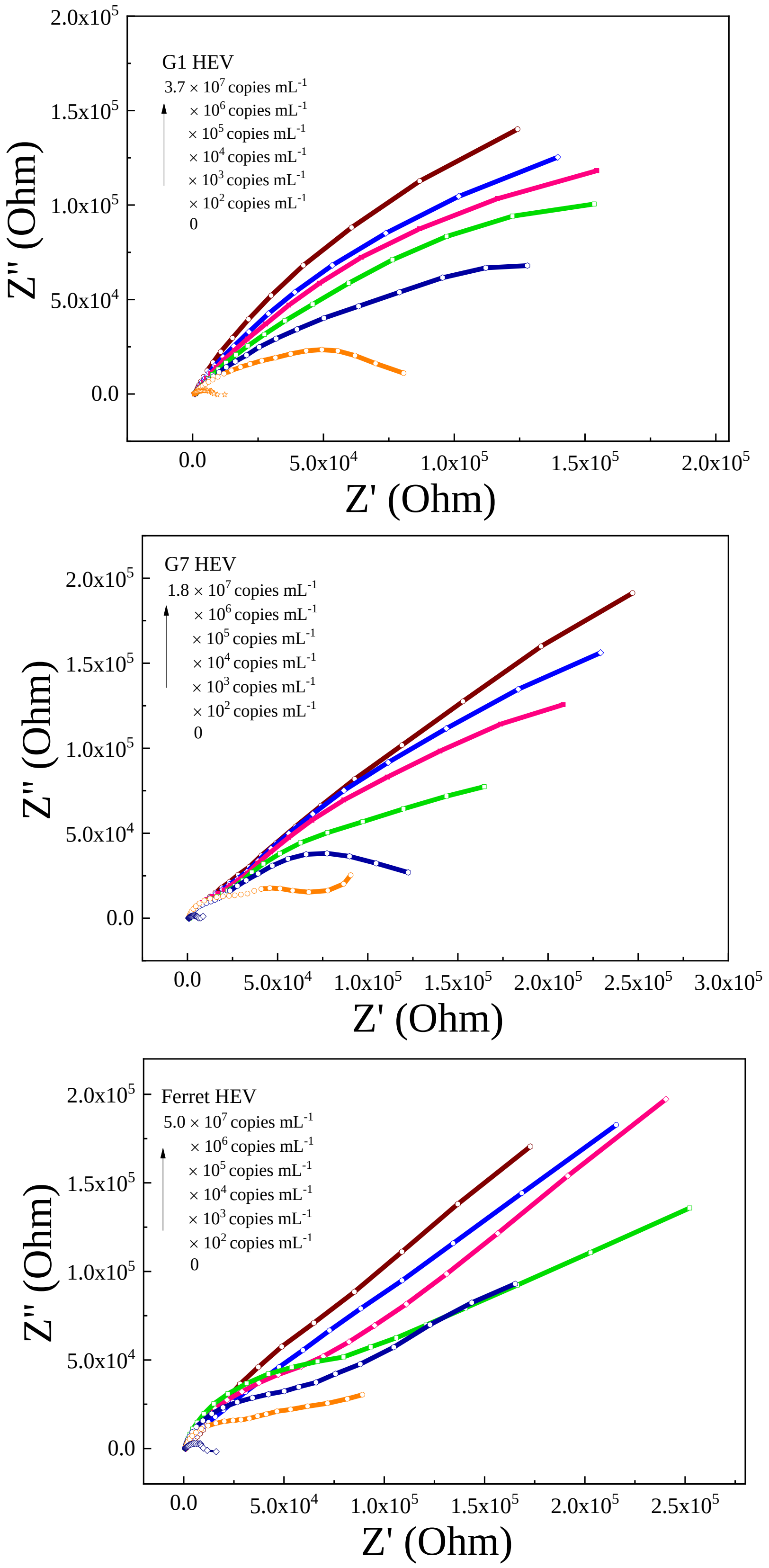

Supplementary Figure 8. Detection of G1, G7 and ferret HEVs from cell culture supernatant.

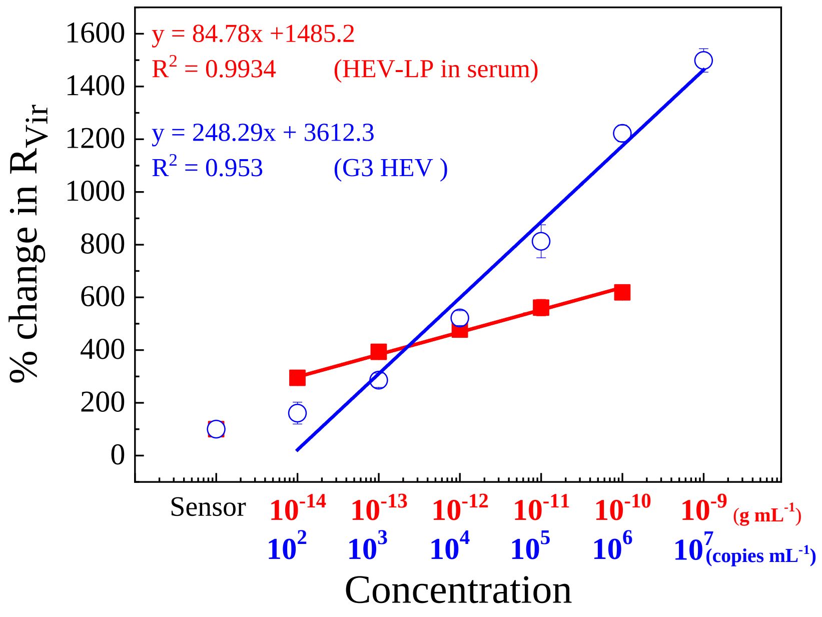

Supplementary Figure 9. Calibration lines of R_{vir} vs. HEV-LP in serum (closed squares) and R_{vir} vs. G3 HEV (open circles) in cell culture supernatant. Error bars represent the standard deviation of three samples.

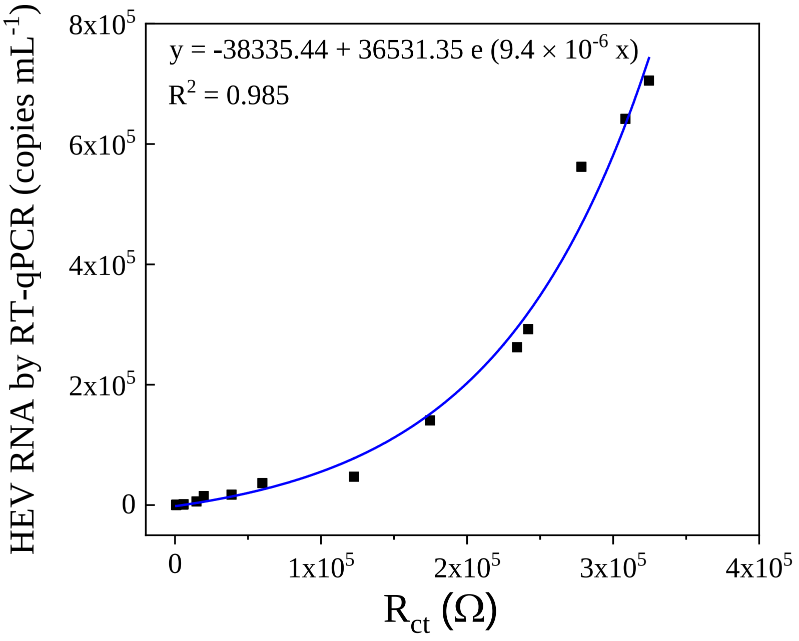

Supplementary Figure 10. Relationship between G7 HEV detection by copy numbers from RT-qPCR and that by the R_{ct} values. G7 HEV samples were from a series of fecal specimens from experimental G7 HEV-infected cynomolgus monkey.

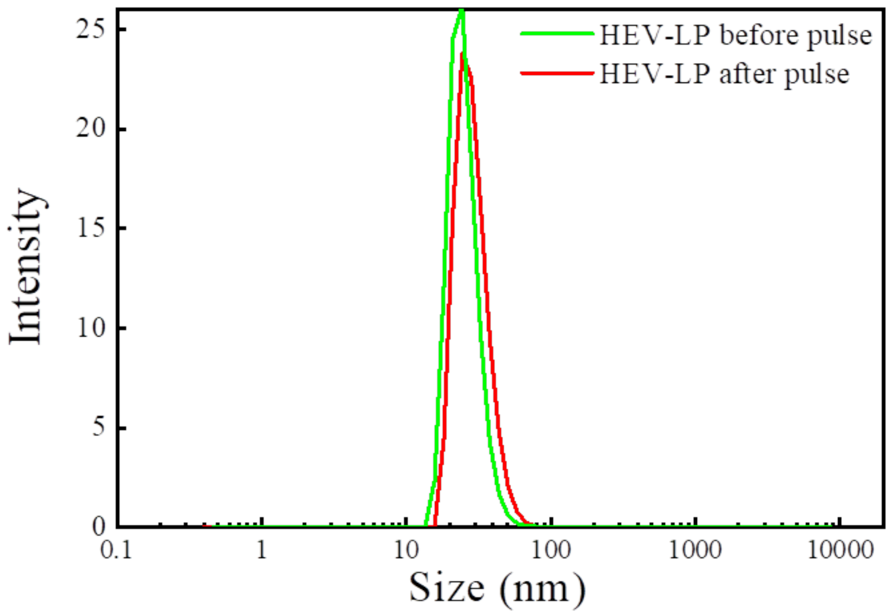

Supplementary Figure 11. DLS measurement of the bare HEV-LP before (green line) and just after (red line) the electrical pulse of +0.8 V.

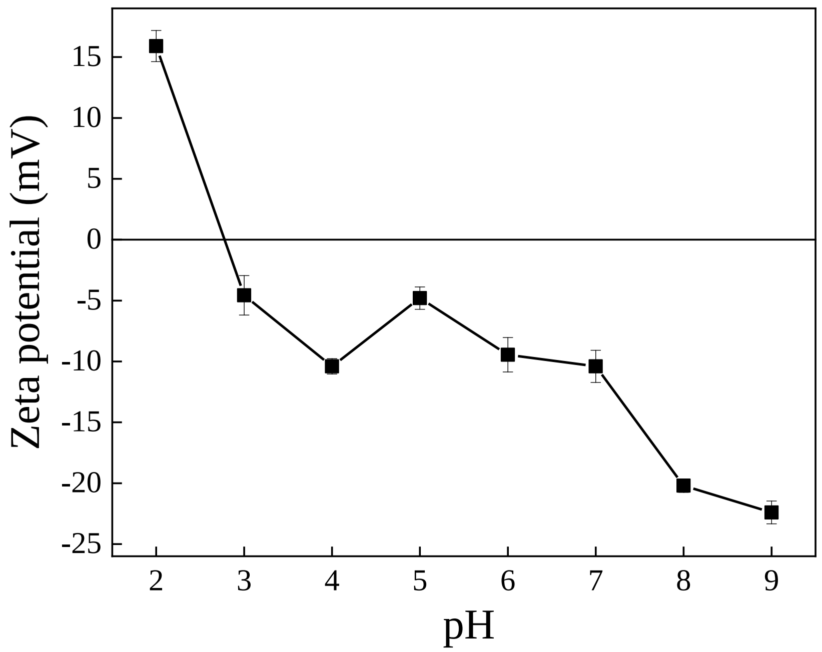

Supplementary Figure 12. Zeta potential measurement of the HEV-LP in various pH conditions. Error bars represent the standard deviation of three samples.

References:

1. Jothikumar N, Cromeans TL, Robertson BH, Meng X, Hill VR. A broadly reactive one-step real-time RT-PCR assay for rapid and sensitive detection of hepatitis E virus. *J Virol Methods* **131**, 65–71 (2006).
2. Li TC *et al.* Production of infectious dromedary camel hepatitis E virus by a reverse genetic system: Potential for zoonotic infection *J Hepatol.* **65**, 1104–1111 (2016).
3. Li TC, *et al.* Production of infectious ferret hepatitis E virus in a human hepatocarcinoma cell line PLC/PRF/5. *Virus Res* **213**, 283-288 (2016).
